# The variability of multisensory processes of natural stimuli in human and non-human primates in a detection task

**DOI:** 10.1371/journal.pone.0172480

**Published:** 2017-02-17

**Authors:** Cécile Juan, Céline Cappe, Baptiste Alric, Benoit Roby, Sophie Gilardeau, Pascal Barone, Pascal Girard

**Affiliations:** 1 Cerco, CNRS UMR 5549, Toulouse, France; 2 Université de Toulouse, UPS, Centre de Recherche Cerveau et Cognition, Toulouse, France; 3 INSERM, Toulouse, France; Centre de neuroscience cognitive, FRANCE

## Abstract

**Background:**

Behavioral studies in both human and animals generally converge to the dogma that multisensory integration improves reaction times (RTs) in comparison to unimodal stimulation. These multisensory effects depend on diverse conditions among which the most studied were the spatial and temporal congruences. Further, most of the studies are using relatively simple stimuli while in everyday life, we are confronted to a large variety of complex stimulations constantly changing our attentional focus over time, a modality switch that can impact on stimuli detection. In the present study, we examined the potential sources of the variability in reaction times and multisensory gains with respect to the intrinsic features of a large set of natural stimuli.

**Methodology/Principle findings:**

Rhesus macaque monkeys and human subjects performed a simple audio-visual stimulus detection task in which a large collection of unimodal and bimodal natural stimuli with semantic specificities was presented at different saliencies. Although we were able to reproduce the well-established redundant signal effect, we failed to reveal a systematic violation of the race model which is considered to demonstrate multisensory integration. In both monkeys and human species, our study revealed a large range of multisensory gains, with negative and positive values. While modality switch has clear effects on reaction times, one of the main causes of the variability of multisensory gains appeared to be linked to the intrinsic physical parameters of the stimuli.

**Conclusion/Significance:**

Based on the variability of multisensory benefits, our results suggest that the neuronal mechanisms responsible of the redundant effect (interactions vs. integration) are highly dependent on the stimulus complexity suggesting different implications of uni- and multisensory brain regions. Further, in a simple detection task, the semantic values of individual stimuli tend to have no significant impact on task performances, an effect which is probably present in more cognitive tasks.

## Introduction

It is widely accepted that multisensory presentation of stimuli (e.g. an auditory stimulus A simultaneous with a visual stimulus V) leads to a behavioural facilitation compared to unisensory presentation (A or V alone) [1]. Many studies have focused on the question of timing, and have demonstrated that reaction times (RT) tend to be faster to multisensory than to unisensory stimuli [2–13]. In the case of visual and auditory multisensory studies, simple detection tasks mostly used simple stimuli (flashes, pure tones…) and the subject has to detect any stimulus as fast as possible [6,7,12]. Cappe and collaborators [14] adapted such paradigms to the rhesus macaque monkey and showed that a simple manual detection task can reveal multisensory integration in this species, like in humans or other species [15–17]. So far, studies on multisensory integration in humans only used ‘simple’ stimuli and/or a limited set of more natural stimuli. In studies with macaque monkeys, the use of more ecological natural stimuli in analogous psychophysical studies is uncommon or also restricted to a limited set of stimuli [18]. The use of natural stimuli seems to be particularly interesting as shown in preferential looking paradigms. For instance, the gaze of human infants and adults is particularly attracted by pictures of conspecific (mostly faces) in a quasi-automatic manner [19]. Another example is illustrated in the study by Dufour and collaborators [20] in which several monkey species as well as humans prefer gazing at conspecific faces. Rhesus macaque monkeys are even able to match a picture of a given conspecific face to the voice of that individual [21,22]. It has also been shown that semantic congruence can facilitate the multisensory integration in explicit identification tasks. For instance, videos coupled with semantically congruent sound are more often brought to awareness [23] and identification of visual natural stimuli is faster when preceded by an auditory congruent prime [24]. Many different factors have been shown to influence multisensory RTs such as spatial and temporal coincidence as well as inverse effectiveness (the weaker the stimuli, the stronger the facilitation) but most of the time these factors have been studied independently [25]. Some recent studies combined some of these factors (i.e. spatial location and effectiveness) using simple stimuli and showed a strong interdependency between these factors in determining behavior and shaping perception [26,27].

In the present study, we performed a simple detection task using various and numerous A, V and AV natural stimuli in both humans and macaque monkeys. This is the first multisensory RT study using such a large set of natural stimuli. Because we used many different stimuli, one could expect a large variability of reaction times to the different exemplars. Our study also addresses the question of possible sources of the variability of reaction times and of multisensory integration. We tested many different factors: semantic congruence, salience, categories, physical parameters of stimuli and modality switch. Trial history is indeed a possible source of RT variability which has been mostly studied under the terms of modality switch. In a task where numerous A, V and AV stimuli are randomly presented, successive trials will randomly switch or keep modality. The influence of trial history on reaction times and multisensory integration has been investigated [28,29] showing that modality switch leads to a cost in terms of reaction times, in particular to unisensory stimuli. This could lead to artificial multisensory integration when results are analysed by race model equations.

Based on the use of a large set of several hundred different natural stimuli, our study revealed a broad range multisensory gain on RTs, with negative and positive gains, that reflects variability regarding stimuli specificity. When all aspects of the stimuli presentation are analysed, from semantic to physical features, the main factors influencing the variability of multisensory gain on RTs appear to be the actual physical parameters of the stimuli mainly linked to the salience, intensity and energy contents.

## Materials and methods

### Ethic statement

All experiments on monkeys were in conformity with the ethical rules of the EEC (EEC, Directive 2010/63/UE). All procedures were in accordance with the Weatherall report, ‘The use of non-human primates in research’ and were fully approved by the local ethical committee named ‘Comité d’Éthique Midi-Pyrénées (MP/03/34/10/09)’.

The study with human subjects was conducted in accordance with the guidelines of the declaration of Helsinki. All subjects signed informed consent and were informed that they could quit the experiments at any time. The experiment was approved by the Inserm ethical committee (IRB00003888- agreement n°14–156).

### Monkey subjects and setup

Two male rhesus macaque monkeys (Macaca mulatta, age: 11 and 4 years old respectively) were used in this study. They were sitting in a primate chair (Crist instruments) placed in the center of a sound attenuated darkened cabin. The head of the monkey was at 57cm from a computer screen (Iiyama, 21”, vision masterpro 512, 75 Hz frame-rate). The loudspeakers (Yamaha MSP3) were positioned on each side of the monitor at the same height as the ears of the monkey. We checked that the loudspeakers did not cause interfering noise to the visual display and reciprocally.

The monkeys were head free but a video camera was used to monitor their behaviour. The chair has an aperture through which the monkey can touch the screen with its dominant hand (determined during the training period).

### Behavioural task

The task was a detection task with the following steps: Each trial started when the monkey pressed a pad located just below the monitor. After a variable delay (100 to 500 ms), a stimulus appeared. As soon as the stimulus appeared, the monkey had 2 s to touch the center of the screen to get a reward. As soon as the monkey touched the screen, the stimulus was turned off and the reward was delivered. If the monkey did not respond within the 2 s period, the stimulus was turned off and an extra delay (1 s) was added to the intertrial interval (600 ms) during which pressing the pad had no effect.

The reaction time corresponded to the time measured from the beginning of the stimulus display to the pad release, as measured by light sensitive diodes.

The monkeys were water restricted during working days and the reward consisted in a few water drops. We carefully measured the weight of the monkeys every training day and gave extra water if needed when they were back in the animal facility.

### Stimuli

The monkeys were naïve to every stimulus used in the study. Stimuli were from two sensory modalities: visual (V, n = 304) or auditory (A, n = 304) which could be combined to form auditory-visual stimuli (AV).

#### Visual stimuli

A large set of natural images from the databank of our laboratory was used. All images were converted to 8-bit BMP grey level pictures. They were normalised in luminance (mean grey value = 128). The size of the images was 13° and they were presented centrally on a grey background (14 cd/m2).

#### Auditory stimuli

A large set of sounds was also taken from the databank of our lab. Sounds were stereo 44 kHz waves normalized in peak intensity and with a 3 ms fading-in. If the sound duration was shorter than 2 s, it was repeated from the beginning until 2 s have elapsed.

#### Category

Images and sounds belonged to 4 categories each split in two subcategories in equal numbers (38 V and 38 A in each subcategory). The categories were: humans (subdivided into males and females subcategories), monkeys (rhesus and non-rhesus), other animals (birds and non-birds), inanimate (objects and landscape). The category pairing for incongruent AV differed from session to session (a picture of a man paired with a monkey call in session 1, another picture of a man coupled with a bird call in session 2).

#### Congruence

AV stimuli were made to be semantically congruent (n = 304) or incongruent (n = 304) at the subcategory level. For instance, a rhesus face could be coupled with a rhesus call (not corresponding to the facial mimic) or a finch coupled with a blackbird call.

#### Salience

Because the salience of each stimuli (V, A or AV) could be high or low, the stimuli database contained 2432 stimuli. High and low saliences of images were due to a change of RMS contrast, at respectively 12% and 2%. Two sound intensities were used: High salience at 71.1 dB and low salience at 54.7 dB.

In each daily session, one stimulus of each 8 subcategory was randomly presented as visual (V), auditory (A) and a combination of both modalities (AV).

The subcategory, the salience and the AV congruence was randomized. Hence in a given session, the monkey saw a total amount of 64 stimuli each randomly repeated 10 times (reaching a total amount of 640 stimulations).

Cortex software (NIMH) in a DOS operating system was used to monitor the behaviour, present the stimuli and acquire the data. The timing of the visual stimuli was checked by a photodiode permanently installed in a corner of the screen. The timing of the auditory stimuli and the synchrony of the AV stimuli was checked on an oscilloscope.

### Human subjects and setup

15 human subjects (6 males and 9 females; mean age 28.2, 11 were right handed) participated in the experiment. All were naïve to the task and the stimuli. The human subjects sat in front of the screen (Trinitron Sony Multiscore E400 19”), their head stabilized by a chin and front device. The eyes were positioned at 40 cm from the screen center. The subjects wore earphones (Sennheiser hd 280 pro). All stimuli were presented in a random order with Eprime 2.0 software. As for monkeys, the timing of the visual stimuli was checked by a photodiode permanently installed in a corner of the screen. The timing of the auditory stimuli and the synchrony of the AV stimuli was checked on an oscilloscope.

### Behavioral task

As for monkeys, the task was a detection task with the following steps. Before starting the experiment, subjects were informed that they would have to press a button as fast as possible whenever an image, a sound or both was played; without trying to distinguish or naming the stimulus.

Before the appearance of each stimulus, a white cross was displayed during a variable period (750 to 1500 ms) to keep the subject attentive to the task. After this delay, the stimulus was displayed during 1 sec. The background of the screen remained permanently grey. The reaction time was defined as the elapsed time from stimulus appearance to button press.

### Stimuli

The human subjects were naïve to every stimulus used in the study. Stimuli were from two sensory modalities: visual (V, n = 72) or auditory (A, n = 72) which could be combined to form auditory-visual stimuli.

#### Visual stimuli

All stimuli were taken from the databank of our laboratory. All images were in color (BMP 24 bits). Image size was 18x20 degrees of visual angle. Luminance and contrast of images have been normalised (Matlab), in equalizing the 3 colour layers of the bitmap images (Red, Green, Blue).

#### Auditory stimuli

The sounds have been taken from the databank of our laboratory. All sounds had an equal duration (800 ms). Sounds were normalized in peak intensity with Adobe Audition 3.0 with a 2 ms fading-in and sound intensity was measured with a sonometer (Roline, RO-1350).

#### Category

Images and sounds belonged to 3 different semantic categories: Faces and voices (men, women and kids), non-faces and non-human sounds (animals, objects, landscapes, vehicles, water, storm) and abstract category. For face and non-face visual stimuli, images were cropped such that faces were centred and animals or objects presented entirely in the absence of a distractor from another category. Visual stimuli from the abstract category were built by randomisation of the phases of the Fourier spectrum of the face stimuli (Matlab) such as faces are no more perceived. Sounds from the abstract category were vocoded versions of the human-voice stimuli (2 channels vocoder) [30]. Each category contained 24 images and 24 sounds (total amount of stimuli was 144).

#### Congruence

AV stimuli were made congruent or incongruent at the category level (n = 144). For example, a voice sound was associated to a non-face image. For the abstract category, we apply the same kind of association and then stimuli were randomized as described just above.

#### Salience

Each of the 72 images was presented under a weak or a strong salience at the center of a computer screen coming up to an amount of 144 visual stimuli. Weak salience images were obtained by lowering the RMS contrast to 7% with respect to high salience condition (RMS = 28%).

Each sound was presented through headphones under 2 conditions: weak intensity at 35dB and strong intensity at 60 dB, coming up to an amount of 144 sounds. AV stimuli were presented as semantically congruent or incongruent at two salience levels (total of 288 AV stimuli).

All stimuli were presented to each subject in 8 blocks of 72 stimuli, each stimulus being presented only once. All conditions (A, V or AV, weak or strong salience, congruent or incongruent, and categories) were randomized. Intertrial interval was randomized between 750 to 2000ms. A pause took place between each block to avoid fatigue.

To summarize, this protocol used 2432 A, V and AV stimuli presented 10 times to 2 monkeys and 576 A, V and AV stimuli presented one single time to 15 subjects. In both species experiments, we manipulated the congruence, the salience and the semantic content of the stimuli. This use of a large set of stimuli and conditions allows us to study the variability in multisensory processes and to determine which factors influence the multisensory integration.

### Behavioral analysis

#### Reaction times and gains

Based on RT distributions, trials for which the RT was faster than 150 ms for monkeys and 100 ms for humans were considered anticipatory, and trials on which the RT was slower than 800 ms for monkeys and humans were considered as failure to comply with task demands. On average in man and monkeys, no more than 5% of the RTs were excluded from the analysis, a result that clearly shows the optimal attentional engagement of the subjects and animals.

We calculated a multisensory gain of reaction times using the following formula:

$$gain=\frac{Umax\;-AV}{Umax}*100$$

where AV is the mean of reaction times in auditory-visual condition and Umax is the mean of reaction times in the best single-modality condition. In the case of monkeys, gains were calculated for each stimulus and then were averaged depending on which effect is studied (salience, congruence, group of stimuli…). Whereas for humans, as each stimulus was presented only once, gains were calculated on all the 15 subjects for each stimulus and then were averaged depending on studied effect.

Two classes of models have been formulated to account for the multisensory gain: race and co-activation models. According to the race model [31], the faster of the two stimuli in competition mediates the behavioral response on any given trial. In this case, probability summation can account for the multisensory gain because the likelihood of either of the two stimuli yielding a fast RT on any given trial is higher than that from either stimulus alone. In contrast, the coactivation model [32] takes into account neuronal multisensory integration. In this case, the threshold to initiate behavioral responses is reached faster with multisensory stimuli leading to faster reaction times than with unisensory stimuli. Along this framework, we tested if the multisensory gain exceeded the facilitation predicted by probability summation using Miller’s so-called “race model” inequality [32]. Miller’s inequality tests whether the probability of a RT of a given speed to a multisensory stimulus is higher than the summed probabilities for an equally fast RT to either unisensory stimulus alone (minus their joint probability under the assumption of complete independence [33]). This entailed calculating the cumulative probability distribution for each condition. The cumulative probability distributions of RTs were first divided into 5% bins on the basis of the range of RTs across stimulus conditions. A model of the probability distribution for each multisensory combination was then calculated. For each 5% bin, the modeled value equals the sum of the probabilities for each component unisensory condition minus their joint probability [i.e., P(RT(A)) + P(RT(V)) − (P(RT(A)) × P(RT(V)))]. The comparison between the modeled probability and the multisensory probability allows determining if there is a violation of the race model meaning integrative processes. On the opposite, no difference means independent sensory processes. We also observed a significant difference but with faster reaction times for the race model which we called an “inverse” violation of race model.

We calculated Miller’s inequality for all stimuli together or grouped by conditions and also in individual stimulus analysis. We first calculated multisensory gains for each stimulus. In the case of individual stimulus analysis, we found positive gains as well as negative gains, so we ranked stimuli by decreasing gains. We then calculated the Miller’s inequality in a sliding window of 30 stimuli, with a step of 3 stimuli. With decreasing gains, the race model was violated for highest gains, satisfied for high gains and inversely violated for lowest and negative gains. This allowed us to define limits of groups of stimuli. Stimuli with higher gains induced a violation of the race model, and were classified in the group 1 of stimuli. The switching from violation to satisfaction of the race model determined the gain limit between group 1 and group 2. Group 2 induced positive gains and satisfied the Race model. The point where stimuli switch from satisfaction to inverse violation of the race model corresponds to the gain limit between group 2 and group 3. Group 3 contained stimuli inducing positive gains and an inverse violation of the Race model. The lower limit between group 3 and group 4 corresponded to a gain equal to 0. Hence, group 4 contained stimuli inducing negative gains and an inverse violation of the Race model. As limits were defined from a sliding window, we verified the global violation, no-violation or inverse violation of each group of stimuli. This method was applied for both monkeys and for humans.

We examined if the reaction time to a given stimulus was affected by the modality switch, i.e. by the modality of the stimulus displayed in the previous trial. For each monkey separately and human subjects put together, reaction times for each modality were split with respect to the previous modality: for instance, all reaction times to AV stimuli that were preceded by A, reaction times to AV stimuli preceded by V and so on.

In a second time, we investigated whether modality switch could have an influence on gains. As multisensory gain was calculated from the fastest unisensory RT (here V RT) and the multisensory RT (AV RT), we explored the modality switch effect only for these two modality conditions. We measured the proportion of trials N in V and AV conditions preceded by A, V and AV stimuli in N-1 trials. These proportions were calculated for each group of stimuli. We obtained a 4*6 matrix (groups*modality combinations). This analysis was done for each monkeys and humans.

#### Impact of stimulus properties

We sought to determine which physical parameters of the images could have an impact on reaction times and gains.

For images, we first determined mean, variance, and signal to noise ratio values of gray level pixels. Second-order statistics were computed from co-occurrence matrices that contain the number of occurrences of two gray-level values, separated by a given pixel distance in a given direction in the image [34]. Among many parameters that can be derived from the matrices, we focused on four of second-order parameters (energy, entropy, inertia, and homogeneity), which we computed across four different orientations (0°, 90°, 180° and 270°) (see Table 1 for the formula). As images used for humans were in color, these second order parameters were calculated for each three levels of color (red, green, blue) and then averaged.

**Table 1:** Formula of physical parameters of sounds and images.

	Parameters	Formula
Sounds	Mean Intensity	$\bar{x}=\frac{1}{N}\sum_{i}^{N}\left(\frac{1}{M}\sum_{i}^{M}I\left(i,j\right)\right)$
	Mean Intensity on 200ms	$\bar{x}200=\frac{1}{200}\sum_{i}^{200}\left(\frac{1}{M}\sum_{j}^{M}I\left(i,j\right)\right)$
	Peak Intensity	$Ipeak200=\text{max}\left(\frac{1}{M}\sum_{j}^{M}I\left(j\right)\right)$
	Time at the peak	$Tpeak200=t\left(\text{max}\left(\frac{1}{M}\sum_{j}^{M}I\left(j\right)\right)\right)$
	RMS	$RMS=\frac{\sqrt{\frac{1}{N}\sum_{ij}{\left(I\left(i,j\right)-\bar{x}\right)}^{2}}}{\bar{x}}$
	Transience	$T=t\left(S1>\frac{\text{max}\left(S1\right)}{2}\right)$
Images	Mean luminance	$\bar{x}=\frac{1}{N}\sum_{i,j}g\left(i,j\right)$
	Variance	$V=\frac{1}{N}\sum_{i,j}{\left(\;g\left(i,j\right)-\bar{x}\right)}^{2}$
	SNR	$SNR=\frac{\bar{x}}{V}$
	Energy	$E=\sum_{i,j}{\left(p\left(i,j\right)\right)}^{2}$
	Entropy	$ENT=-\sum_{i}\sum_{j}p\left(i,j\right)\text{log }p\left(i,j\right)$
	Inertia	$INT=\sum_{i}\sum_{j}{\left(i-j\right)}^{2}p\left(i,j\right)$
	Homogeneity	$HOM=\sum_{i,j}\frac{1}{1+{\left(i-j\right)}^{2}}p\left(i,j\right)$

For the acoustic analysis, we examined low level parameters, namely the mean intensity of sounds on the full duration, and other parameters on the first 200ms of the sounds: mean intensity, intensity and time of the peak, root mean square (RMS) and transience (see Table 1 for the formula). We also examined the richness of sounds based on the model of Chi and collaborators [35] which is inspired by psycho-acoustical and neurophysiological findings in the auditory system. This model generates spectrograms of the sounds and a parameter called the ratescale. Ratescale is a 4D matrix which is a combination between descriptors of the spectrotemporal modulation (Rate, 2D) and the distribution of energy in the frequency channels (scale, 2D). For example, the human voice has a high scale because it consists of specific small bands of frequencies that are energy rich, whereas white noise has a low scale because of an evenly distributed energy across large bands of frequency. For analytic purposes, the average of the 4d matrix was extracted according to the spatial dimensions to compute the rate scale over time. All ratescale computations have been computed with NSL toolbox in Matlab [35]. We have put together sounds of each group of stimuli (as defined above according to the gains and race-model) and calculated the mean ratescale across time.

#### Statistical analysis

Because the distribution of reaction times and gains were not normal for any tested condition (Shapiro-Wilk test), we used a Kruskal-Wallis test (for reaction times, gains, physical parameters) for multiple comparisons, Friedman test (for ratescale values) for multiple paired comparisons and post hoc were done using a Mann-Whitney test or a Wilcoxon test with the Bonferroni correction. We used a Mann-Whitney test (for reaction times and for gains) and Wilcoxon test (for Race model comparisons) for simple comparisons. We used a Pearson Chi square test to compare the proportion of congruent and incongruent stimuli between different stimuli groups defined above. Pearson Chi square test was also used to compare the proportion of weak and strong stimuli and to compare the proportion of stimuli of each category in the different groups.

## Results

### Effect of modality on RTs

**Fig 1:**
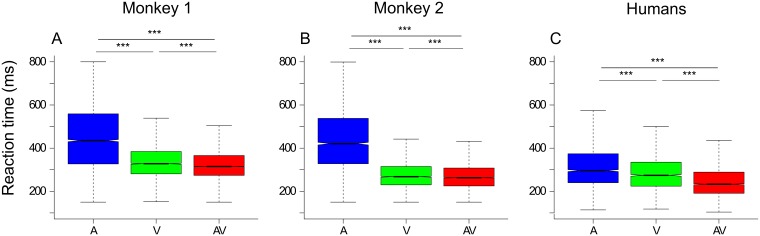
Reaction time data for monkey 1 (A), for monkey 2 (B) and for humans (C). Blue box plots correspond to auditory (A), green to visual (V) and red to auditory—visual (AV) stimuli. The box and horizontal bar within represent the interquartile range and the median of RT, respectively. The whiskers extend to the most extreme data point, which is no more than 1.5 times the interquartile range from the box. The notch approximates a 95% confidence interval for the median. Significance is reported using asterisks depending on the P value: * for p<0.05, ** for p<0.01 and *** for p<0.001.

We first studied the effect of the modality on reaction times. As reported in previous studies, we found that, for both monkeys and for human subjects, the modality of the stimulus had a strong effect on reaction times (RT). We indeed found significant shorter reaction times in AV condition and longer reaction times in A condition (Fig 1 and S1 Table). To characterize this modality effect, we calculated the multisensory gain according to the formula described in the method section. Because the auditory modality was the slowest, the mean multisensory gain was computed from the V and AV reaction times. The mean multisensory gain was rather low for monkeys (2.9 for monkey 1 and 0.64 for monkey 2) but markedly higher for humans (12.6). We also examined whether the race model could explain our results. When the race model was applied to the overall set of trials, we unexpectedly observed that the race model was satisfied. The validation of the race model was present for both monkeys and the human data meaning that multisensory integration was not responsible for the shortening in bimodal RTs.

### Effect of conditions and categories

#### Reaction times

We then examined salience, congruence and category effects, as well as their cross effects, on reaction times. We expected shorter RTs for stimuli in strong salience as well as for semantically congruent stimuli and for stimuli of conspecific categories. We actually observed the expected effect of salience, more precisely, we found, in both monkeys and in humans, significant shorter RTs to strong salience stimuli. However, we did not observe any influence of category or of congruence on uni- or bimodal RTs. All these results were found for all cross-comparisons between salience, congruence and category (see S2 Table for all statistical analysis).

#### Gains

**Fig 2:**
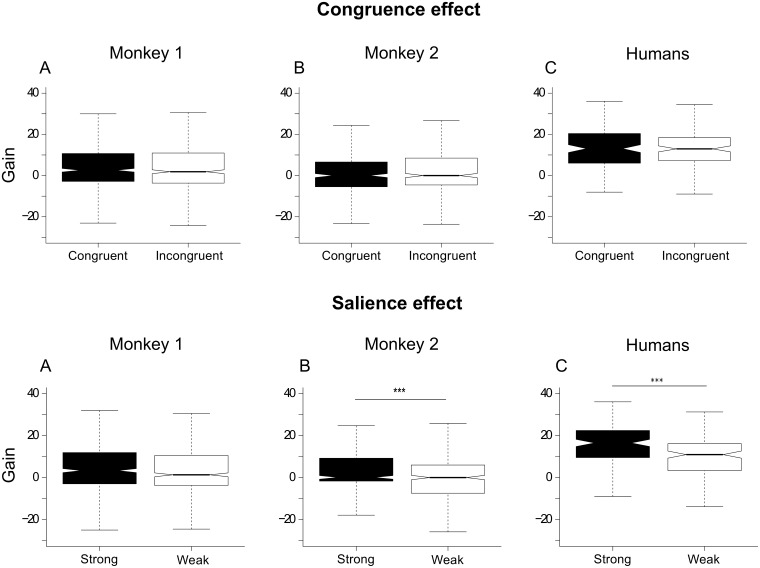
Effect of congruence (upper part) and salience (lower part) on multisensory gains. Same convention as in Fig 1 for the box plots. Significance is reported using asterisks depending on the P value: * for p<0.05, ** for p<0.01 and *** for p<0.001.

Our study aimed to understand the behavioral gain caused by multisensory information and to understand rules underlying this process. We explored whether salience, congruence and category could influence behavioral gains. We expected higher gains in weak salience condition according to the principle of inverse effectiveness. However, we found the opposite effect of salience and no effect of congruence and category (Fig 2 and S3 Table). More precisely, we found significant higher gains for strong salience in monkey 2 and in humans but not in monkey 1. This was found for all cross-comparisons.

### Effect of stimuli on gains

**Fig 3:**
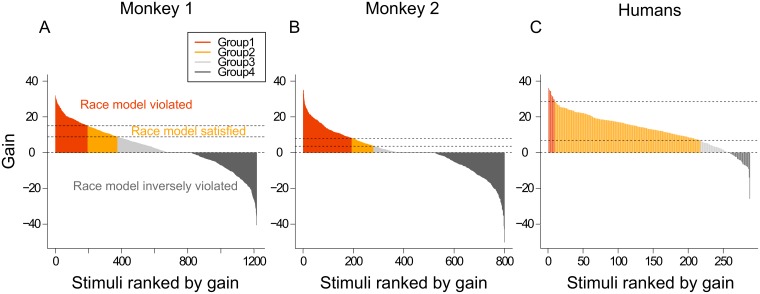
Multisensory gain for each stimulus, ranked by gain, for the 3 subject species. Group 1 (orange) corresponds to stimuli which induce positive gain and race model violation, group 2 (yellow) to stimuli which induce positive gain and satisfy race model, group 3 (light grey) to stimuli which induce positive gain and violate the race model inversely and group 4 (dark grey) to stimuli with negative gain and violate the race model inversely.

As described previously, we examined the Miller’s inequality in all conditions and we never found race model violation, neither in monkey, nor in humans. These results contradict previous studies that were mostly conducted with simple stimuli such as bars and dots for visual stimuli and noises and tones for auditory stimuli. Because we used more complex natural stimuli to put our study in a more ecological context, we wanted to verify whether the complexity of our stimuli could lead to different multisensory processes and whether it could result in variability. This analysis aims at examining our results at the level of individual stimuli. First, we calculated the multisensory gain obtained for each different stimulus in each crossed salience or congruence conditions. Then we ranked all the stimuli by decreasing gains values. This simple individual analysis revealed that some stimuli induced large multisensory gains while other stimuli elicited no gain or even negative gains (Fig 3). We found this kind of distribution in both monkeys and in humans. This result was unexpected because negative gains have never been reported in previous studies. We examined whether the race model was violated or satisfied at the level of individual trials. From the ranked distribution of gains, we calculated the Miller’s inequality in a 30-stimuli sliding window with a step of 3 stimuli (see Materials and methods section for more details), for each monkey and for humans (Fig 3 and S4 Table). This allowed us to define 4 groups of stimuli according to the race model violation. The first group of stimuli corresponded to the bimodal stimuli resulting in the highest positive gains and as well as a violation of the race model. The second group corresponded to positive multisensory gains but with a validation of the race model. The third group corresponded to positive gains and an inverse violation of the race model, meaning that a significant difference was found between bimodal condition and the probability summation but with shorter RTs for the race model than in multisensory condition. The fourth group corresponded to negative gains and an inverse violation of the race model.

We investigated what can explain the difference of processes leading to these four groups (S5 Table). We first examined the influence of salience, congruence and category by looking at their distribution across the different groups. First, we compared the repartition of stimuli salience inside each group with a Pearson Chi square test. We found the same result as with global gains, i.e. an effect of salience but not of congruence or category. More precisely, the group 1 leading to the highest gains and a violation of the race model, included more stimuli (73.3% on average) with high salience. Inversely, group 4 corresponding to negative gains and an inverse violation of the race model, included statistically more stimuli with low salience (66% on average). This statistical difference was observed in monkey 2 and in humans. The cross comparison was not performed for this analysis (S5 Table). As mentioned previously, the analysis of the stimuli repartition across the 4 groups according to the category and the semantic congruence did not reveal a statistical difference.

### Role of the physical parameters of the stimuli

As we used many various natural stimuli, we hypothesized that intrinsic parameters specific to the stimuli could influence the gain values and the violation of the race model. To test that, we computed several physical parameters from both visual and auditory stimuli.

#### Sounds

We investigated the mean intensity of sounds over the full duration and some other parameters of sounds between 0 and 200ms, namely, mean intensity, intensity and time of the peak, RMS and transience (S6 Table, see Materials and methods for more information). There was some indication that parameters like intensities of the sound (full duration and first 200ms) and RMS contribute to the differences between groups for monkey 2 and humans. For monkey 2, we found an effect of the mean intensity between 0 and 200ms on groups of stimuli, with significantly lower values for the third group compared to the second and the fourth. In this monkey, the RMS between 0 and 200ms also differed between groups of stimuli, with higher RMS values for the group 4 compared to the groups 1 and 3. The mean intensity on the full duration of the sound differed between groups of stimuli, but this difference failed to pass the post-hoc Bonferroni correction. However, we did not find any difference linked to any of these parameters between groups of stimuli for monkey 1 (S6 Table).

For humans, we found an effect of these three parameters and also of the peak intensity between 0 and 200ms of sounds. It appeared that, in humans, group 1, providing highest gains and a violation of race model, contained sounds with higher intensity, peak and RMS values. All these parameters seem to be linked to the gain values as they change in the same way. For the mean intensity of the full sound duration, we obtained a difference only between group 1 and group 4. For the mean intensity and the RMS between 0 and 200ms, the group 4 had lower values compared to groups 1 and 2. For the peak intensity between 0 and 200ms, groups 1 and 4 differed from all other groups, with higher values for group 1 and lower values for group 4 (S6 Table).

To conclude, the main features that characterized the group 1 sounds in humans are higher intensity, peak and RMS. Low values of these parameters characterized group 4 stimuli.

**Fig 4:**
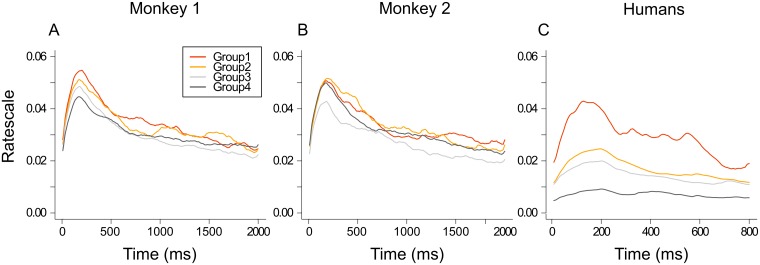
Ratescale values of sounds as a function of time for the four groups of stimuli. Group 1: positive gain and violation of race model, group 2: positive gain and race model satisfied, group 3: positive gain and inverse violation of race model and group 4: negative gain and inverse violation of race model. Groups are described in Fig 3. Note that there are differences between group 4 versus all other groups of stimuli for humans, which is not found for monkeys.

We also studied the ratescale because it represents the richness of sounds taking into account the spatiotemporal information and the energy of frequencies (see Materials and methods for a more detailed description). We compared the ratescale between different groups of stimuli and it appeared that it could explain, at least in part, the different groups of stimuli for humans, but not for monkeys (Fig 4, statistical data not shown). Indeed, for humans, we found a significant difference of ratescale values of group 1 compared to every other group all along the sound duration. As shown on Fig 4, stimuli providing the highest gains (group 1) corresponded to highest ratescale values and stimuli providing the lowest gains (group 4) had the lowest ratescale values. It seems that group 2 had higher ratescale values than group 3 although this did not reach statistical significance. This tendency was also observed for monkey 1 and monkey 2.

#### Images

For images, we examined the possible role of 7 physical parameters of visual stimuli (first and second order, see Materials and methods): mean, variance, signal-noise ratio, homogeneity, energy, inertia, and entropy. For monkey 2, the energy was significantly higher in group 1 than in group 2 of stimuli. We also found an effect of entropy which is significantly higher in group 2 compared to other groups. However, for monkey 1, we did not find any difference linked to any of these parameters between groups of stimuli. For humans, group 1 contained stimuli with lower energy values than in group 4 (S7 Table).

### Effect of modality switch

**Fig 5:**
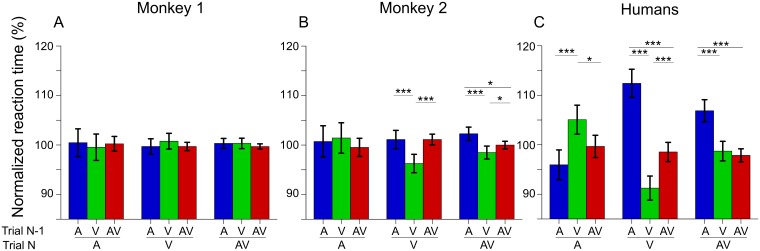
Percentage of normalized reaction time for different combinations of successive modalities. Auditory (A) N trials are plotted in the left part of the figures, visual (V) in the center and auditory-visual (AV) in the right part. N trials are sorted by N-1 trials’ modality, with auditory on the left in blue, visual in the center in green and auditory-visual on the right in red. Once sorted, N-1 reaction times are divided by the median reaction time of all N trials for the corresponding modality. For example, for the three leftmost bars, the data was normalized by dividing by the median reaction time of all auditory trials. Bars and error bars represent respectively median and 95% confidence interval of the median. Significance is reported using asterisks depending on the P value: * for p<0.05, ** for p<0.01 and *** for p<0.001.

Because we used randomized trials with different stimulus modalities, some successive trials will remain in the same modality and some will switch modality. It has been shown that a modality switch can slow down reaction times whereas keeping the same modality speeds up reaction times [29,36–40]. We hypothesized that such effect of modality switch on reaction times could influence the multisensory gains. We first determined whether and how the modality switch influenced reaction time. As we used three different modalities (A, V, AV), we obtained 9 different modality combinations of two successive trials that we compared for each monkeys and for humans trials. For humans, we found an effect of modality switch on reaction times (Fig 5, see S8 Table for statistical results). In unisensory conditions, reaction times were shorter when a modality was preceded by the same modality (V followed by V in the next trial for example) and longer when a modality is preceded by the opposite modality (A followed by V). In multisensory condition, reaction times were shorter when a stimulus is preceded by a visual or AV stimulus and longer when preceded by an auditory stimulus.

For monkey 2, as for humans, reaction times were shorter when a visual stimulus was preceded by a visual stimulus than by an auditory or auditory-visual stimulus. In AV condition, reaction times were longer when stimuli were preceded by an auditory stimulus, like in humans. A striking point in this monkey is that shorter reaction times to AV stimuli were produced by preceding visual stimuli and not by AV stimuli. We did not observe any effect of the switch for auditory stimuli in monkey 2. In monkey 1, we did not find any effect of modality switch on reaction times.

Because we found an effect of modality switch on RT, at least in monkey 2 and humans, we investigated whether modality switch could have an effect on gains. As gain is calculated from the fastest unisensory RT which is the Visual modality, we measured the proportion of trials in V and AV conditions preceded by A, V or AV stimuli and this was done for each group of stimuli. We performed a Pearson Chi square test (S9 Table) on the 4*6 matrix for each monkeys and humans. The result of this analysis showed no difference between stimuli groups, meaning that the modality switch did not influence the gain value neither it influenced the violation of the race model.

## Discussion

In this study, we have used a large set of several hundred different natural stimuli to explore multisensory mechanisms in a simple detection task in humans and monkeys. Such an approach allowed us to analyse whether different aspects intrinsic to the stimuli, from low level to cognitive content, may impact on responsiveness and multisensory interactions.

We reproduced a global redundant signal effect [8,41] in both species. Our study revealed a large range of gains at the stimuli level, from strongly positive to strongly negative gains, which has never been reported in previous studies. The gain variability reflects probably diverse multisensory processes from co-activation or race model and inhibition processes between sensory modalities. Our results reveal that one of the main sources of the response variability is the trial history and more specifically we show that the changes in trial modalities strongly modulate the RTs to uni- or bimodal stimuli. Other sources of variability for multisensory processes originate from physical parameters of stimuli, such as salience of stimuli, energy of images and intensity, peak and RMS of sounds.

Altogether, our data revealed that the multisensory integration, as expressed by the violation of the race model, should also be considered in a deleterious aspect, depending on the type of bimodal stimuli presented. Such phenomenon is probably a general mechanism as it applied to both human and non-human primates.

### Multisensory processes and MS gains

The fastest response to AV stimuli is a redundant signal effect that could be simply explained by a probability summation which is often described as a race model between A and V channels. Consequently, it is usually considered that a true multisensory integration is present when reaction times to bimodal stimuli are faster than predicted by probability summation [7,12,14,42]. We used the quite conservative model to test the Miller inequality [33] and found that globally, reaction times did not violate the race model. Compared to previous studies based on a more liberal model, our result could originate from such difference between models [29,43]. Because in our study we used a more naturalistic design with many different complex natural stimuli, it allows us to compare stimulus driven multisensory patterns and thus to consider another origin, namely that the variability of the race model violation could be stimulus-dependent.

We observed that, for both rhesus and humans, multisensory gains to our large set of stimuli present an unexpected broad distribution, from the well-known strong positive gains to strong negative gains for which AV reaction times were clearly slower than the fastest unimodal stimuli. No other study has provided evidence of such negative gains, because generally gains are calculated globally by pooling all the data together. Indeed, when we calculated a global MS gain we observed, as largely reported, a positive mean value of MS gain. In order to quantify the multisensory integration with respect to gains, we defined groups of stimuli according to the fact that reaction times induced, or not, a violation of the race model. Interestingly, we found an inversely violated race model which has never been reported before. In that case, there is a significant probability to respond with a slower RT to a multimodal stimulus than to probability summation. In that situation, the multisensory integration leads to very low gains, null gains or even costs instead of positive gains. The fact that numerous gains were negative could not be explained by momentary lapses of attention or disengagement from the task, firstly because subjects had to engage voluntarily to complete the task. Secondly, this was also not related to trials belonging to the beginning (‘warming-up’) or end of the sessions (‘fatigue’). Among factors that could explain the variability of gains, we discuss below the possible roles of modality switch and physical parameters of the stimuli.

### Modality switch

One of our main results concerns the effect of modality switch upon multisensory integration. The modality switch has been shown to affect reaction times in active tasks in which subjects need to identify or detect a visual stimulus preceded by a sound or reciprocally [28,44–49]. This effect is inescapably related to sequential effects, the situation where successive trials of the same modality lead to faster and better performances (repetition priming) than when the modalities alternate [29,36–40]. This phenomenon characterizes the situation in which subjects focusing their attention to a modality are slowed down or make more errors when they need to switch their attention to another modality to complete a task [45,46,50,51].

Firstly, we examined the trial history or more precisely the modality switch effect on reaction times in our multimodal natural stimulus detection task. We observed that reaction times to unimodal stimuli were shorter when the same modality was presented in the preceding trial and longer when there was a modality switch. This was verified in humans and also in monkey 2 for visual stimuli. Importantly, the modality switch effect has rarely been studied with bimodal stimuli in a simple detection task [29,47]. Generally it is considered that the bimodal stimuli could not be primed or affected because they contain both modalities. While we observed the expected cost to switch of unimodal stimuli, the RTs to bimodal stimuli were also affected. Indeed, the RTs to AV stimuli were also slowed down when preceded by auditory stimuli, this was true for monkey 2 and humans. Another interesting point is that, unlike humans, RTs to AV stimuli were faster when preceded by V than by AV for monkey 2. These observations could be related to the visual dominance/auditory penalty in primates [46,52–56]. Furthermore, the visual dominance might be more accentuated in macaques as one could infer from the difficulties to train these animals to pure auditory tasks [57–59,50]. The exacerbated visual dominance of the macaques could lead to different modality switch effects on bimodal stimuli between species.

As we observed different multisensory patterns (gains and multisensory processes) at the stimulus level, we investigated whether the modality switch effect found on reaction times could explain this variability. Interestingly, Gondan and collaborators [28] have yet pointed out that switch costs in reaction times could artificially lead to race model violation in a simple detection task in human subjects. These authors explained this phenomenon by the fact that the RTs to unimodal stimuli are lengthened by the switch unlike those to bimodal stimuli. They observed a decrease of the magnitude of the redundant effects but still observed violations restricting the analysis of race model to unswitched stimuli only. Our analysis on the proportion of switched trials versus repeated trials in the 4 groups of stimuli according to race model violations showed that the groups did not differ in terms of ratio of switches. This result tends to proscribe any effect of modality switch on MS gain and indicates that some configurations of AV stimuli lead to strong gains or costs, independently of the switch effects. We interpret these results such that more probably the multisensory integration benefit could be high enough to counteract the deleterious effect of switch.

The precise neuronal mechanisms by which multisensory stimulation could counteract the effect of trial history remain to be studied. Recent studies plead in favour of a synchronization/desynchronization mechanism to explain the modality switch effect. Firstly, in studies in which subjects actively switch in a task from the visual to the auditory modality, alpha desynchronizations are observed over the parieto-occipital cortex whereas alpha synchronisation would be present if the modality is repeated [60,61]. When the task is an active task, a top down control mechanism from the frontal lobes is involved interacting with lower sensory or polysensory areas [60,62,63]. One function of the top-down controlled alpha synchronisation is to favour attentional processing while ignoring distractors, in parallel with gamma waves synchronisation [62,64–66,67]. The phase synchronisation is indeed crucial as the perception level is highly dependent on the phase at which the stimulus is presented [68].

However, the aforementioned works concern cases of endogenous switch of attention in which subjects are instructed to make a switch or attentively follow its consequence. In our study, although the subjects pay globally attention to the task to detect a stimulus, they do not have to care about the switches or repeated modality trials (exogenous attention). Nevertheless, modality switches in the present study could be considered as distracting episodes because they imped RTs. This being said, multisensory processing could be efficient because we observed that gains are not affected by trial history; in other terms, a strong multisensory gain could be observed despite the presence of switches that could drag attention away from the previous modality. Brunet et al [69] have shown that repetition of (visual) stimuli lead to increase of gamma synchronization in the monkey visual cortex (within V1 and between V1 and V4) with respect to trials with no repetition. This observation echoes the well-known mechanisms of phase reset of gamma activity caused by multisensory stimulation [70–78]. Hence, in bottom-up situation involving exogenous attention (modality switch), we propose that multisensory stimulation can be mediated through synchronisation mechanisms which are analogous to the ones operating in top down voluntary attention control, in order to efficiently counteract the distracting effect of switches. The neuronal structures specifically involved in this process are unknown and should be the goal of further investigations. One possible candidate is the pulvinar that could be viewed as a switchboard operator involved in synchronizing areas according to attention priorities [79]. The fact that the pulvinar may be involved both in top down [79]and bottom up attentional controls [80] or even (unconscious) drive of attention to potential dangers [81] makes this structure an interesting candidate to study the effect of switch onto multisensory integration in comparing top-down and bottom up conditions.

### Intrinsic features of stimuli

As this variability of the multisensory patterns in stimuli detection has never been reported before, we sought to understand what factors could explain it. The starting point to discuss the variability is the fact that we found an advantage for strongly salient stimuli for race model violation. Many behavioral or neurophysiological studies have shown that multisensory integration is more efficient when stimuli are close to detection threshold, a phenomenon usually referred to as inverse effectiveness [82–84]. Unexpectedly, we observed that the multisensory gain was stronger for salient stimuli. We supposed that visual contrast and sound intensity of our stimuli were not close enough to the perceptual threshold to induce inverse effectiveness. As we used many various natural stimuli, it was virtually impossible to determine perceptual thresholds.

Because the salience derives from the intrinsic physical parameters of the stimuli, this prompted us to examine some physical features of the stimuli other than simple visual contrast and sound intensity to study their possible relationship with gain and with the engaged multisensory process.

For images, several parameters (first and second order statistics) were computed. These parameters measure the number of grey-level transitions in an image, when the transitions are numerous, because of the resulting clutter, some images may be considered as less salient in terms of content (camouflage [85]). We found an effect of homogeneity as measured by energy and entropy. For humans, highest gain stimuli (Group1) have lower energy values, meaning that they had the lowest homogeneity (many grey level transitions). In visual categorization tasks, the most homogeneous images led to the best performances in terms of RT [86,87]. As a consequence, we could expect that longer RTs to low energy visual stimuli may have contributed to higher MS gains, a result that is in line with the stimulus repartition in group 1.

Concerning the sound properties, we examined the role of many low level parameters. We found that some of them (mean intensity value, RMS, intensity of peak) varied according to multisensory gain. The direct link between those parameter values and the group of gains was not very clear in macaques unlike in humans, for whom the gains were clearly dependent on mean intensity, peak value and RMS computed in the first 200 ms of the sound stimuli. This later result could be related to the study of Chen and Spence [88], in which the authors considered that the accumulation of information over time could have an influence on reaction times, such as the faster the accumulation, the faster the reaction time.

We also examined the ratescale that represents the richness of sounds. High ratescale values are typical of sound signals like speech that contain a lot of variation of energy over time. Although it is known that macaques integrate faces and voices in communication situations [89] and brain responses can be sensitive to ratescale [90,91], macaque RTs did not seem to be dependent on ratescale values in our detection task. Interestingly, in human subjects, the ratescale values were significantly higher for stimuli leading to violations of the race model (group 1). However, the category analysis did not reveal an effect of conspecific stimuli on the distinction of the different group of multisensory gains. In spite of such apparent discrepancy, it could be that the human propensity for visuo-auditory integration of faces and speech predisposes a preferential fast treatment of stimuli with high ratescale values.

This later observation leads to logically examine a possible semantic influence on MS integration. The experiment was designed such that half multimodal stimuli were semantically congruent and half incongruent and such that stimuli belonged to different semantic categories. Unfortunately, we never found any semantic effect (congruence or category) on reaction times or even on multisensory gains for both species. In the seminal work on multisensory integration done at neuronal level, the concept of congruence in the temporal [92] and spatial [93] domain has been proposed as pivotal, and some studies have also shown that the semantic information can influence the behavior. Indeed, a semantic congruence improved performances in detection [94], categorization [95], identification [23] [24] and also in memory tasks [96,97]. Moreover, certain semantic categories such as conspecifics are processed in a preferred manner as it has been shown in preferential looking paradigms in humans and monkeys [19] [20]. Furthermore, it seems that primates can have access to semantic information without focused attention to displayed stimuli [98–100]. The semantic effect found in these studies were revealed mainly on humans subjects, but as macaques seem to have also access to a certain level of semantic understanding of multisensory stimuli [21,22,101,102], we expected to observed a semantic effect for both humans and monkeys.

One hypothesis to explain our results could be that a simple detection task, contrary to a more cognitive task, does not require a semantic processing of the stimuli content to induce behavioral responses. The modulation of multisensory processes with the cognitive load has already been observed when comparing detection versus discrimination tasks [103,104]. A specific multisensory integration for the conspecific category would then occur only in active tasks where subjects need to associate faces with speech or monkey calls to extract identity information (like gender, age, prosody, emotional status) during communication [105–107].

## Conclusion

In general, multisensory integration is studied with a limited set of simple stimuli despite the fact that in everyday life, we are confronted to a large variety of stimuli with modalities constantly changing over time. Altogether, our data point to a variability of gains firstly depending on a variety of intrinsic physical parameters of natural stimuli.

Further, our results highlight that the violation of the race model is not a systematic process that can account for multisensory interactions when performing a simple detection task. Co-activation process of sensory information can take place from low perceptual stages to higher premotor or cognitive stages. Here we revealed that such neuronal mechanism is affected by the trial history and the physical features of the stimuli. Such results open a fascinating field of investigations on the rules that trigger such neuronal mechanisms in which brain oscillations should probably play an important role.

Although general conclusions could be drawn concerning multisensory information with large sets of stimuli, we would add the cautious note to take variability into consideration as different stimuli configurations could lead to enhancement or suppression in the same behavioral study just as it does in electrophysiological studies. In our study no single feature of the stimuli, either low physical or high semantic level, can explain the differences in terms of reaction times and MS gains. A complex combination of parameters cannot be excluded but is particularly difficult to reveal because of the number of possible combinations with bimodal stimuli. However, both parameters linked to clutter in visual scenes (perceptively low salient) and rich content of sound (like interspecific communication), along with some factors including the interplay between trial history and brain oscillation phases, could influence multisensory integration. This point to the relevance of our data for further studies, in particular electrophysiological, that would investigate underlying mechanisms of the variability in multisensory processing using a large set of natural stimuli.

## Supporting information

S1 Table Statistical results of modality effect on reaction times. (PDF)

S2 Table Statistical results of salience, congruence and category effect on reaction times. (PDF)

S3 Table Statistical results of salience, congruence and category effect on multisensory gains. (PDF)

S4 Table Description of the four groups of stimuli in terms of race-model violation. (PDF)

S5 Table Statistical results of salience, congruence and category effect on different groups of stimuli. (PDF)

S6 Table Statistical results of effect of sound parameters on different groups of stimuli. (PDF)

S7 Table Statistical results of effect of image parameters on different groups of stimuli. (PDF)

S8 Table Statistical results of effect of modality switch on reaction times. (PDF)

S9 Table Statistical results of effect of modality switch on gains. (PDF)
