# Supplementary material for: The variability of multisensory processes of natural stimuli in human and non-human primates in a detection task
Source: PLoS One. 2017 Feb 17;12(2):e0172480. doi: 10.1371/journal.pone.0172480 (PMC5315309; doi:10.1371/journal.pone.0172480)
Supplement: S1 Table — (PDF) [file pone.0172480.s001.pdf]

		Test	DF	Parameter	P corrected	
Monkey 1	A vs V vs AV	Kruskal-Wallis	2	2677	<0.001	***
	A vs V	Mann-whitney	1	19681871	<0.001	***
	A vs AV	Mann-whitney	1	62460795	<0.001	***
	V vs AV	Mann-whitney	1	50934676	<0.001	***
Monkey 2	A vs V vs AV	Kruskal-Wallis	2	3670	<0.001	***
	A vs V	Mann-whitney	1	9168203	<0.001	***
	A vs AV	Mann-whitney	1	27797679	<0.001	***
	V vs AV	Mann-whitney	1	19355620	<0.001	***
Humans	A vs V vs AV	Kruskal-Wallis	2	772	<0.001	***
	A vs V	Mann-whitney	1	2566200	<0.001	***
	A vs AV	Mann-whitney	1	2761600	<0.001	***
	V vs AV	Mann-whitney	1	3262300	<0.001	***
