# Supplementary material for: The variability of multisensory processes of natural stimuli in human and non-human primates in a detection task
Source: PLoS One. 2017 Feb 17;12(2):e0172480. doi: 10.1371/journal.pone.0172480 (PMC5315309; doi:10.1371/journal.pone.0172480)
Supplement: S2 Table — (PDF) [file pone.0172480.s002.pdf]

		Test	DF	Parameter	P value	
Monkey 1	Salience	Mann Whitney	1	67433000	<0.001	***
	Congruence	Mann Whitney	1	131370000	0.79	
	Category	Kruskal-Wallis	3	3.2	0.36	
	Salience x Congruence	Kruskal-Wallis	3	353.2	<0.001	***
	CS vs CW	Mann Whitney	1	28887000	<0.001	***
	IS vs IW	Mann Whitney	1	28765000	<0.001	***
	CW vs IW	Mann Whitney	1	31788000	0.92	
	CS vs IS	Mann Whitney	1	33912000	0.63	
	Salience x Category	Kruskal-Wallis	7	297.9	<0.001	***
	Weak Category	Kruskal-Wallis	3	1.6	0.66	
	Strong Category	Kruskal-Wallis	3	1.7	0.65	
	MonkeySalience	Mann Whitney	1	4202100	<0.001	***
	Animal Salience	Mann Whitney	1	4190400	<0.001	***
	Human Salience	Mann Whitney	1	4226200	<0.001	***
	Inanimate Salience	Mann Whitney	1	4239100	<0.001	***
	Congruence x Category	Kruskal-Wallis	7	4.4	0.73	
	Salience x Congruence x Category	Kruskal-Wallis	15	358	<0.001	***
Monkey 2	Salience	Mann Whitney	1	25636000	<0.001	***
	Congruence	Mann Whitney	1	51691000	0.61	
	Category	Kruskal-Wallis	3	6.1	0.11	
	Salience x Congruence	Kruskal-Wallis	3	115.2	<0.001	***
	CS vs CW	Mann Whitney	1	11779000	<0.001	***
	IS vs IW	Mann Whitney	1	11720000	<0.001	***
	CW vs IW	Mann Whitney	1	12647000	0.88	
	CS vs IS	Mann Whitney	1	13195000	0.59	
	Salience x Category	Kruskal-Wallis	7	111.5	<0.001	***
	Weak Category	Kruskal-Wallis	3	0.85	0.84	
	Strong Category	Kruskal-Wallis	3	9.2	<0.05	*
	Monkey Salience	Mann Whitney	1	1604600	<0.001	***
	Human Salience	Mann Whitney	1	1614100	<0.001	***
	Animal Salience	Mann Whitney	1	1577300	<0.001	***
	Inanimate Salience	Mann Whitney	1	1612000	<0.001	***
	Congruence x Category	Kruskal-Wallis	7	8.2	0.32	
	Salience x Congruence x Category	Kruskal-Wallis	15	127.4	<0.001	***
Humans	Salience	Mann Whitney	1	173	<0.05	*
	Congruence	Mann Whitney	1	111	0.97	
	Category	Kruskal-Wallis	2	0.87	0.65	
	Salience x Congruence	Kruskal-Wallis	3	12.8	<0.01	**
	CS vs CW	Mann Whitney	1	51	<0.01	**
	IS vs IW	Mann Whitney	1	53	<0.05	*
	CW vs IW	Mann Whitney	1	113	1	
	CS vs IS	Mann Whitney	1	112	1	
	Salience x Category	Kruskal-Wallis	5	21.8	<0.001	***
	Weak Category	Kruskal-Wallis	2	2.3	0.32	
	Strong Category	Kruskal-Wallis	2	0.11	0.94	
	Abstract Salience	Mann Whitney	1	165	<0.05	*
	Human Salience	Mann Whitney	1	178	<0.01	**
	Non-human Salience	Mann Whitney	1	180	<0.01	**
	Congruence x Category	Kruskal-Wallis	5	2	0.85	

Salience x Congruence x Category	Kruskal-Wallis	11	43.2	<0.001	***

NB : CS = Congruent Strong salience; CW = Congruent Weak salience; IS = Incongruent Strong salience; IW = Incongruent Weak salience
