# Supplementary material for: The variability of multisensory processes of natural stimuli in human and non-human primates in a detection task
Source: PLoS One. 2017 Feb 17;12(2):e0172480. doi: 10.1371/journal.pone.0172480 (PMC5315309; doi:10.1371/journal.pone.0172480)
Supplement: S3 Table — (PDF) [file pone.0172480.s003.pdf]

		Test	DF	Parameter	P value	
Monkey 1	Salience	Mann Whitney	1	174260	0.08	
	Congruence	Mann Whitney	1	187150	0.7	
	Category	Kruskal-Wallis	3	5.6	0.1	
	Salience x Congruence	Kruskal-Wallis	3	3.2	0.4	
	Salience x Category	Kruskal-Wallis	7	17.8	0.08	
	Congruence x Category	Kruskal-Wallis	7	6.3	0.5	
	Salience x Congruence x Category	Kruskal-Wallis	15	14	0.53	
Monkey 2	Salience	Mann Whitney	1	68598	<0.001	***
	Congruence	Mann Whitney	1	76654	0.3	
	Category	Kruskal-Wallis	3	6.2	0.1	
	Salience x Congruence	Kruskal-Wallis	3	13.3	<0.01	**
	CS vs CW	Mann Whitney	1	22778	<0.05	*
	IS vs IW	Mann Whitney	1	22990	<0.01	**
	CW vs IW	Mann Whitney	1	19290	0.54	
	CS vs IS	Mann Whitney	1	1904	0.38	
	Salience x Category	Kruskal-Wallis	7	20.3	<0.01	**
	Weak Category	Kruskal-Wallis	3	1.6	0.7	
	Strong Category	Kruskal-Wallis	3	6.8	0.08	
	Monkey Salience	Mann Whitney	1	3569	0.6	
	Human Salience	Mann Whitney	1	4012	<0.05	*
	Animal Salience	Mann Whitney	1	4991	<0.001	***
	Inanimate Salience	Mann Whitney	1	4584	0.31	
	Congruence x Category	Kruskal-Wallis	7	7.4	0.4	
	Salience x Congruence x Category	Kruskal-Wallis	15	21.4	0.1	
Humans	Salience	Mann Whitney	1	6774	<0.001	***
	Congruence	Mann Whitney	1	10584	0.8	
	Category	Kruskal-Wallis	2	3.1	0.2	
	Salience x Congruence	Kruskal-Wallis	3	26	<0.001	***
	CS vs CW	Mann Whitney	1	3489	<0.001	***
	IS vs IW	Mann Whitney	1	3488	<0.001	***
	CW vs IW	Mann Whitney	1	2582	0.97	
	CS vs IS	Mann Whitney	1	2737	0.6	
	Salience x Category	Kruskal-Wallis	5	29.3	<0.001	***
	Weak Category	Kruskal-Wallis	2	1.1	0.6	
	Strong Category	Kruskal-Wallis	2	2.7	0.3	
	Abstract Salience	Mann Whitney	1	677	<0.001	***
	Human Salience	Mann Whitney	1	770	<0.01	**
	Non-human Salience	Mann Whitney	1	798	<0.01	**
	Congruence x Category	Kruskal-Wallis	5	4	0.5	
	Salience x Congruence x Category	Kruskal-Wallis	11	31.7	<0.001	***

NB : CS = Congruent Strong salience; CW = Congruent Weak salience; IS = Incongruent Strong salience; IW = Incongruent Weak salience
