# Supplementary material for: The variability of multisensory processes of natural stimuli in human and non-human primates in a detection task
Source: PLoS One. 2017 Feb 17;12(2):e0172480. doi: 10.1371/journal.pone.0172480 (PMC5315309; doi:10.1371/journal.pone.0172480)
Supplement: S4 Table — (PDF) [file pone.0172480.s004.pdf]

		GP 1	GP 2	GP 3	GP 4
Race model		Violated	Non-violated	Inversely violated	Inversely violated
Monkey 1	Gain	15 to 31.9	8.8 to 15	0 to 8.8	-40.5 to 0
	Number	195	180	298	543
Monkey 2	Gain	8 to 35	3.6to 8	0 to 3.6	-50.1 to 0
	Number	193	86	98	423
Humans	Gain	28.5 to 36	6.9 to 28.5	0 to 6.9	-25.7 to 0
	Number	9	208	42	29

Gain: limits of gain values for each given group

Number: number of stimuli in each group
