# Supplementary material for: The variability of multisensory processes of natural stimuli in human and non-human primates in a detection task
Source: PLoS One. 2017 Feb 17;12(2):e0172480. doi: 10.1371/journal.pone.0172480 (PMC5315309; doi:10.1371/journal.pone.0172480)
Supplement: S5 Table — (PDF) [file pone.0172480.s005.pdf]

		Test	DF	Parameter	P value	
Monkey 1	Salience	Pearson Chi test	3	5.4	0.1	
	Congruence	Pearson Chi test	3	0.69	0.9	
	Category	Pearson Chi test	9	8.6	0.5	
	Salience x Congruence	Pearson Chi test	9	8.3	0.5	
Monkey 2	Salience	Pearson Chi test	3	8.2	<0.05	*
	Congruence	Pearson Chi test	3	8.2	0.5	
	Category	Pearson Chi test	9	12.2	0.2	
	Salience x Congruence	Pearson Chi test	9	11.3	0.3	
Humans	Salience	Pearson Chi test	3	17.5	<0.001	*
	Congruence	Pearson Chi test	3	3.5	0.3	
	Category	Pearson Chi test	6	5.8	0.4	
	Salience x Congruence	Pearson Chi test	9	23.8	<0.01	**
