# Supplementary material for: The variability of multisensory processes of natural stimuli in human and non-human primates in a detection task
Source: PLoS One. 2017 Feb 17;12(2):e0172480. doi: 10.1371/journal.pone.0172480 (PMC5315309; doi:10.1371/journal.pone.0172480)
Supplement: S6 Table — (PDF) [file pone.0172480.s006.pdf]

		Test	DF	Parameter	P corrected	
Monkey 1	Whole intensity	Kruskal-Wallis	3	2.2	0.54	
	Intensity	Kruskal-Wallis	3	2.2	0.52	
	Peak intensity	Kruskal-Wallis	3	2.9	0.41	
	Peak time	Kruskal-Wallis	3	5.1	0.17	
	RMS	Kruskal-Wallis	3	3.2	0.36	
	Transience	Kruskal-Wallis	3	4.6	0.21	
Monkey 2	Whole intensity	Kruskal-Wallis	3	9.5	<0.05	*
	G1 vs G2	Mann Whitney	1	7359	1.08	
	G1 vs G3	Mann Whitney	1	9981	3.8	
	G1 vs G4	Mann Whitney	1	35952	0.079	
	G2 vs G3	Mann Whitney	1	4698	0.79	
	G2 vs G4	Mann Whitney	1	17344	4.7	
	G3 vs G4	Mann Whitney	1	17721	0.12	
	Intensity	Kruskal-Wallis	3	15.3	<0.01	**
	G1 vs G2	Mann Whitney	1	6869	0.2	
	G1 vs G3	Mann Whitney	1	10334	1.9	
	G1 vs G4	Mann Whitney	1	35800	0.064	
	G2 vs G3	Mann Whitney	1	5124	<0.05	*
	G2 vs G4	Mann Whitney	1	18044	4.6	
	G3 vs G4	Mann Whitney	1	16558	<0.01	**
	Peak intensity	Kruskal-Wallis	3	7.5	0.057	
	Peak time	Kruskal-Wallis	3	2.1	0.55	
	RMS	Kruskal-Wallis	3	16.6	<0.001	***
	G1 vs G2	Mann Whitney	1	6929	0.25	
	G1 vs G3	Mann Whitney	1	10264	2.2	
	G1 vs G4	Mann Whitney	1	35130	<0.05	*
	G2 vs G3	Mann Whitney	1	5072	0.063	
	G2 vs G4	Mann Whitney	1	17438	5	
	G3 vs G4	Mann Whitney	1	16459	<0.01	**
	Transience	Kruskal-Wallis	3	5.3	0.15	
Humans	Whole intensity	Krukal-Wallis	3	8.2	<0.05	*
	G1 vs G2	Mann Whitney	1	1184	1	
	G1 vs G3	Mann Whitney	1	249	1.1	
	G1 vs G4	Mann Whitney	1	206	0.063	
	G2 vs G3	Mann Whitney	1	4646	3.9	
	G2 vs G4	Mann Whitney	1	3830	0.097	
	G3 vs G4	Mann Whitney	1	757	0.76	
	Intensity	Krukal-Wallis	3	16.4	<0.001	***
	G1 vs G2	Mann Whitney	1	1272	0.39	
	G1 vs G3	Mann Whitney	1	283	0.19	
	G1 vs G4	Mann Whitney	1	216	<0.05	*
	G2 vs G3	Mann Whitney	1	5096	0.81	
	G2 vs G4	Mann Whitney	1	4160	<0.01	**
	G3 vs G4	Mann Whitney	1	762	0.68	
	Peak intensity	Krukal-Wallis	3	22.8	<0.001	***
	G1 vs G2	Mann Whitney	1	1332	0.17	
	G1 vs G3	Mann Whitney	1	314	<0.05	*
	G1 vs G4	Mann Whitney	1	232	<0.01	**
	G2 vs G3	Mann Whitney	1	5336	0.24	
	G2 vs G4	Mann Whitney	1	4301	<0.001	***

G3 vs G4	Mann Whitney	1	762	0.67	
Peak time	Krukal-Wallis	3	7.8	0.051	
RMS	Krukal-Wallis	3	20.2	<0.001	***
G1 vs G2	Mann Whitney	1	1304	0.26	
G1 vs G3	Mann Whitney	1	298	0.071	
G1 vs G4	Mann Whitney	1	222	<0.05	*
G2 vs G3	Mann Whitney	1	5316	0.27	
G2 vs G4	Mann Whitney	1	4238	<0.01	**
G3 vs G4	Mann Whitney	1	753	0.83	
Transience	Krukal-Wallis	3	8.4	<0.05	*
G1 vs G2	Mann Whitney	1	714	1.4	
G1 vs G3	Mann Whitney	1	103	0.18	
G1 vs G4	Mann Whitney	1	78	0.46	
G2 vs G3	Mann Whitney	1	3520	0.19	
G2 vs G4	Mann Whitney	1	2459	0.69	
G3 vs G4	Mann Whitney	1	625	5.9	
