# Supplementary material for: The variability of multisensory processes of natural stimuli in human and non-human primates in a detection task
Source: PLoS One. 2017 Feb 17;12(2):e0172480. doi: 10.1371/journal.pone.0172480 (PMC5315309; doi:10.1371/journal.pone.0172480)
Supplement: S7 Table — (PDF) [file pone.0172480.s007.pdf]

		Test	DF	Parameter	P corrected	
Monkey 1	Mean	Kruskal-Wallis	3	4	0.3	
	Variance	Kruskal-Wallis	3	5.4	0.1	
	SNR	Kruskal-Wallis	3	3.6	0.3	
	Energy	Kruskal-Wallis	3	0.9	0.8	
	Entropy	Kruskal-Wallis	3	0.4	0.9	
	Inertia	Kruskal-Wallis	3	5	0.2	
	Homogeneity	Kruskal-Wallis	3	0.4	0.9	
Monkey 2	Mean	Kruskal-Wallis	3	3.3	0.35	
	Variance	Kruskal-Wallis	3	0.63	0.89	
	SNR	Kruskal-Wallis	3	3.3	0.35	
	Energy	Kruskal-Wallis	3	9.8	<0.05	*
	G1 vs G2	Mann Whitney	1	10170	<0.01	**
	G1 vs G3	Mann Whitney	1	9380	0.85	
	G1 vs G4	Mann Whitney	1	28803	0.16	
	G2 vs G3	Mann Whitney	1	3272	<0.01	
	G2 vs G4	Mann Whitney	1	10261	0.058	
	G3 vs G4	Mann Whitney	1	14627	0.23	
	Entropy	Kruskal-Wallis	3	9.4	<0.05	*
	G1 vs G2	Mann Whitney	1	6500	<0.01	**
	G1 vs G3	Mann Whitney	1	9635	0.85	
	G1 vs G4	Mann Whitney	1	25175	0.27	
	G2 vs G3	Mann Whitney	1	5139	<0.05	*
	G2 vs G4	Mann Whitney	1	13599	<0.05	*
	G3 vs G4	Mann Whitney	1	12592	0.31	
	Inertia	Kruskal-Wallis	3	4.3	0.23	
	Homogeneity	Kruskal-Wallis	3	6.9	0.074	
Humans	Mean	Kruskal-Wallis	3	7.7	0.051	
	Variance	Kruskal-Wallis	3	2.1	0.55	
	SNR	Kruskal-Wallis	3	6.3	0.096	
	Energy	Kruskal-Wallis	3	12.6	<0.01	**
	G1 vs G2	Mann Whitney	1	3260	0.06	
	G1 vs G3	Mann Whitney	1	3066	0.1	
	G1 vs G4	Mann Whitney	1	1465	<0.05	*
	G2 vs G3	Mann Whitney	1	2021	5.8	
	G2 vs G4	Mann Whitney	1	1020	3.7	
	G3 vs G4	Mann Whitney	1	936	3.5	
	Entropy	Kruskal-Wallis	3	5.4	0.14	
	Inertia	Kruskal-Wallis	3	5	0.18	
	Homogeneity	Kruskal-Wallis	3	6.7	0.082	
