# Supplementary material for: The variability of multisensory processes of natural stimuli in human and non-human primates in a detection task
Source: PLoS One. 2017 Feb 17;12(2):e0172480. doi: 10.1371/journal.pone.0172480 (PMC5315309; doi:10.1371/journal.pone.0172480)
Supplement: S8 Table — (PDF) [file pone.0172480.s008.pdf]

	Trial N	Trial N - 1	Test	DF	Parameter	P corrected	
Monkey 1	AV		Kruskal-Wallis	2	0.33135	0.85	
	A		Kruskal-Wallis	2	1.1778	0.55	
	V		Kruskal-Wallis	2	3.7348	0.15	
Monkey 2	AV		Kruskal-Wallis	2	17.911	<0.001	***
		AV_V	Mann Whitney	1	2991800	0.021	*
		AV_A	Mann Whitney	1	2924600	0.037	*
		A_V	Mann Whitney	1	1083800	<0.001	***
	A		Kruskal-Wallis	2	0.86378	0.65	
	V		Kruskal-Wallis	2	17.102	<0.001	***
		AV_V	Mann Whitney	1	813280	<0.001	***
		AV_A	Mann Whitney	1	731250	1.47	
		A_V	Mann Whitney	1	274780	<0.001	***
Humans	AV		Kruskal-Wallis	2	39.9	<0.001	***
		AV_V	Mann Whitney	1	1165500	2.52	
		AV_A	Mann Whitney	1	1283600	<0.001	***
		A_V	Mann Whitney	1	643960	<0.001	***
	A		Kruskal-Wallis	2	14.267	<0.001	**
		AV_V	Mann Whitney	1	283860	0.018	*
		AV_A	Mann Whitney	1	278740	0.46	
		A_V	Mann Whitney	1	123240	<0.001	***
	V		Kruskal-Wallis	2	104.36	<0.001	***
		AV_V	Mann Whitney	1	259090	<0.001	***
		AV_A	Mann Whitney	1	332210	<0.001	***
		A_V	Mann Whitney	1	188060	<0.001	***
