# Supplementary material for: The variability of multisensory processes of natural stimuli in human and non-human primates in a detection task
Source: PLoS One. 2017 Feb 17;12(2):e0172480. doi: 10.1371/journal.pone.0172480 (PMC5315309; doi:10.1371/journal.pone.0172480)
Supplement: S9 Table — (PDF) [file pone.0172480.s009.pdf]

	Test	DF	Parameter	P corrected
Monkey 1	Pearson Chi test	15	5.7	0.98
Monkey 2	Pearson Chi test	15	11.5	0.72
Humans	Pearson Chi test	15	13.1	0.59
